# CD11b Signaling Prevents Chondrocyte Mineralization and Attenuates the Severity of Osteoarthritis

**DOI:** 10.3389/fcell.2020.611757

**Published:** 2020-12-18

**Authors:** Driss Ehirchiou, Ilaria Bernabei, Véronique Chobaz, Mariela Castelblanco, Thomas Hügle, Alexander So, Li Zhang, Nathalie Busso, Sonia Nasi

**Affiliations:** ^1^Service of Rheumatology, Department of Musculoskeletal Medicine, Centre Hospitalier Universitaire Vaudois, University of Lausanne, Lausanne, Switzerland; ^2^Department of Physiology, Center for Vascular and Inflammatory Diseases, University of Maryland School of Medicine, Baltimore, MD, United States

**Keywords:** CD11b, integrin, calcium-containing crystals, osteoarthritis, animal model, chondrocyte mineralization, cartilage calcification, human cartilage

## Abstract

Osteoarthritis (OA) is a progressive joint disease that is strongly associated with calcium-containing crystal formation (mineralization) by chondrocytes leading ultimately to cartilage calcification. However, this calcification process is poorly understood and treatments targeting the underlying disease mechanisms are lacking. The CD11b/CD18 integrin (Mac-1 or αMβ2), a member of the beta 2 integrin family of adhesion receptors, is critically involved in the development of several inflammatory diseases, including rheumatoid arthritis and systemic lupus erythematosus. We found that in a collagen-induced arthritis, CD11b-deficient mice exhibited increased cartilage degradation compared to WT control animals. However, the functional significance of CD11b integrin signaling in the pathophysiology of chondrocytes remains unknown. CD11b expression was found in the extracellular matrix and in chondrocytes in both healthy and damaged human and murine articular cartilage. Primary murine CD11b KO chondrocytes showed increased mineralization when induced *in vitro* by secondary calciprotein particles (CPP) and quantified by Alizarin Red staining. This increased propensity to mineralize was associated with an increased alkaline phosphatase (Alp) expression (measured by qRT-PCR and activity assay) and an enhanced secretion of the pro-mineralizing IL-6 cytokine compared to control wild-type cells (measured by ELISA). Accordingly, addition of an anti-IL-6 receptor antibody to CD11b KO chondrocytes reduced significantly the calcification and identified IL-6 as a pro-mineralizing factor in these cells. In the same conditions, the ratio of qRT-PCR expression of collagen X over collagen II, and that of Runx2 over Sox9 (both ratio being indexes of chondrocyte hypertrophy) were increased in CD11b-deficient cells. Conversely, the CD11b activator LA1 reduced chondrocyte mineralization, Alp expression, IL-6 production and collagen X expression. In the meniscectomy (MNX) model of murine knee osteoarthritis, deficiency of CD11b led to more severe OA (OARSI scoring of medial cartilage damage in CD11b: 5.6 ± 1.8, in WT: 1.2 ± 0.5, *p* < 0.05, inflammation in CD11b: 2.8 ± 0.2, in WT: 1.4 ± 0.5). In conclusion, these data demonstrate that CD11b signaling prevents chondrocyte hypertrophy and chondrocyte mineralization *in vitro* and has a protective role in models of OA *in vivo*.

## Introduction

Osteoarthritis (OA) is a long-term chronic degenerative and disabling joint disease (Sen and Hurley, 2020) for which no effective curative disease modifying treatment is available. A wide range of current treatments only lessen the symptom but not the underlying condition. Understanding the pathological mechanisms and the signaling pathways controlling OA development and progression could provide novel targets that would improve the treatment of this disorder (Huang et al., 2018; Kim et al., 2018).

OA is characterized by progressive loss of articular cartilage, osteophyte formation, synovial inflammation and calcification in joint structures such as cartilage (Ea et al., 2011; Loeser et al., 2012; Frallonardo et al., 2018; Yan et al., 2020). Calcium-containing crystals, which encompass basic calcium phosphate (BCP) and calcium pyrophosphate dihydrate crystals (CPPD), have been demonstrated to be of etiologic importance in OA. Indeed they were shown to actively trigger inflammatory, catabolic and oxidant responses in joint cells such as synovial fibroblasts, macrophages, osteoclasts and chondrocytes (Fuerst et al., 2009; Nasi et al., 2016b; Bertrand et al., 2020; Yan et al., 2020). However, the receptors and signaling pathways modulating ectopic calcification in OA joints only start to be elucidated (Castelblanco et al., 2020).

Cell-surface receptors, such as integrins, mediate interaction between chondrocytes and the extracellular matrix (ECM) components, orchestrating its physiological turn-over. Integrins are composed of different combinations of α- and β-subunits, most of which have been found expressed in chondrocytes. In joint physiology, integrins have been demonstrated to play key roles in chondrogenic differentiation (Varas et al., 2007; LaPointe et al., 2013; Song and Park, 2014) and chondrocyte survival (Loeser, 2014). In joint pathology, lack of integrin signaling has shown to mediate the catabolic reactions responsible for cartilage matrix degradation, ultimately leading to OA (Lapadula et al., 1997; Forsyth et al., 2002; Almonte-Becerril et al., 2014; Loeser, 2014; Tian et al., 2015). For instance, mice deficient in β1 integrins exhibit disorganized articular cartilage, reduced mobility and increased OA changes (Raducanu et al., 2009). On the other hand, cartilage ECM components such as fibronectin and collagens, are known ligands of integrins. In fact, Forsyth et al. have reported that α5β1 integrin mediates matrix degradation induced by fibronectin fragments in cultured chondrocytes (Forsyth et al., 2002).

CD11b/CD18 (Mac-1 or α_*M*_β_2_) is a member of the beta 2 integrin family of adhesion receptors (Rosetti and Mayadas, 2016; Schittenhelm et al., 2017). CD11b serves as a signaling regulator and its deficiency has been implicated in the development of inflammatory diseases, such as systemic lupus erythematosus (SLE), lupus nephritis and rheumatoid arthritis (Ehirchiou et al., 2007; Reed et al., 2013; Stevanin et al., 2017; Khan et al., 2018). In SLE, genetic variants in the human *ITGAM* gene, which encodes for CD11b, have been strongly associated with susceptibility to the disease (Nath et al., 2008). Furthermore, Dahdal et al. (2018) have reported a positive correlation between disease activity and serum calcification-propensity, as measured by a novel functional blood test (T_50_) that is able to measure the time for spontaneous formation of crystalline calcium phosphate deposits. It has also been revealed that CD11b is involved in MyD88/TRIF pathway in SLE, by decreasing IFN-α,β and NF-κB transcribed cytokines, namely interleukin-6 (IL-6) (Faridi et al., 2017), which can promote calcium-containing crystal formation (Nasi et al., 2016b). An additional evidence of the effect of CD11b on reducing the pro-mineralizing IL-6, is in a mouse model of collagen-induced arthritis, where our laboratory reported that CD11b KO mice exhibited higher IL-6 serum levels as compared with those of WT control animals (Stevanin et al., 2017).

However, the role of CD11b integrin in calcification has not yet been examined. Based on these observations, we hypothesized that CD11b integrin signaling might be involved in the process of cartilage calcification. Here, we found that both murine and human chondrocytes expressed CD11b integrin. Furthermore, our results indicate that CD11b deficiency in chondrocytes disrupted articular cartilage homeostasis by enhancing chondrocytes calcification, via increased IL-6 production and a switch toward hypertrophic cell differentiation, overall leading to cartilage degradation. Finally, our results identified a newly CD11b-dependent pathway that may be a target in the future development of a successful therapeutic approach in OA.

## Materials and Methods

### Primary Murine Chondrocytes Preparation and Cultures

Articular chondrocytes were isolated from C57BL/6J and CD11b KO mice as described previously (Nasi et al., 2016a). Cells were cultured at a density of 3.5 × 10^4^ cells/cm^2^ for 7 days in complete DMEM (10% fetal bovine serum (FBS), 1% Penicillin Streptomycin). Medium was changed every 2–3 days. Cells were detached with trypsin-EDTA (Amimed) and counted for stimulation experiments, all performed in complete DMEM medium, with LA1 20uM (ChemBridge) dissolved in dimethyl sulfoxide (DMSO) or equivalent quantity of the vehicle as control, added 30 min before the mineralization experiment. Chondrocytes were stimulated with secondary calciprotein particles (CPP) for 4, 6, or 24 h, or in BGjb medium (10% FBS, 1% Penicillin Streptomycin) with 50 μg/mL ascorbic acid and 20 mM β-glycerol phosphate for 14 days. Cell monolayers were stained with Mmp13 rabbit polyclonal antibody (Abcam 39012) and quantified by ImageJ as percentage of positively stained cells over total cells in each field. Chondrocytes were stimulated with 10 μg/ml rat anti-mouse IL-6R (15A7, Bio X Cell) monoclonal antibody or irrelevant IgG2b antibody control.

### Calciprotein Particles Preparation

CPP were generated by preparing 20 ml DMEM containing 10% FBS, 1 mmol/L CaCl2, 3.5 mmol/L inorganic phosphate (1.36 mmol/L NaH2PO4 and 2.14 mmol/L Na2HPO4) (Aghagolzadeh et al., 2016). After 7 days incubation at 37°C, the suspension was centrifuged for 3 h at 4500 rpm and 4°C, and the pellet containing CPP was resuspended in 1 ml DMEM and stored at −20°C.

### LDH Measurement

Measurement of lactate dehydrogenase (LDH) as a means of cytotoxicity assay was performed with using CytoTox-ONE Homogeneous Membrane Integrity Assay (Promega) according to the manufacturer’s instructions. Values were calculated as percentage of cytotoxicity using the following formula: LDH release (%) = [(value in sample) — (background)]/[(value in Triton X-100-treated sample) — (background)] x100.

### Crystal Detection From Chondrocyte Cultures

Chondrocytes were treated with CPP (50 μg/ml calcium) for 24 h or calcifying medium for 24 h, then washed in PBS and fixed in 10% formaldehyde. Crystals were quantified with Alizarin Red staining with Adobe^®^ Photoshop^®^ after image binarization and related pixel counts. Calcium content was quantified by the QuantiChromTM Calcium Kit (BioAssay Systems) by reading absorbance at 612 nm using the Spectramax M5e reader (Molecular Devices).

### ELISA Measurements

Supernatants were collected from stimulation experiments, and assayed using murine or human IL-6 and murine MCP-1 ELISA kits (eBioscience) following the manufacturer’s protocol. Results were read at 450 nm by Spectramax M5e (Molecular devices). Synovial fluid was obtained from 24 OA patients at time of joint replacement (average age 69 years old ± 10), and CD11b evaluated in these biological samples by human CD11b ELISA (LSBio, LifeSpan BioSciences, Inc.).

### Real Time Quantitative PCR Analysis

Cells were treated with TRIzol (500 μl for 1 million cells) and RNA was extracted (RNA Clean and Concentrator 5-Zymoresearch) and reverse transcribed (Superscript II- Invitrogen^TM^). Quantitative Real Time PCR (qRT-PCR) was performed with gene specific primers (Table 1) using the LightCycler^®^ 480 system (Roche Applied Science). Normalization was performed against Gapdh reference gene, and fold increase of transcripts was calculated against control cells.

**TABLE 1:** Gene primers (forward and reverse) sequences for RT-qPCR analysis (*m*, murine; *h*, human).

Primer gene	Forward primer (5′→ 3′)	Reverse primer (3′→ 5′)
*mSox9*	AAG ACT CTG GGC AAG CTC TGG A	TTG TCC GTT CTT CAC CGA CTT CCT
*mColl2*	ACA CTT TCC AAC CGC AGT CA	GGG AGG ACG GTT GGG TAT CA
*mRunx2*	GGG AAC CAA GAA GGC ACA GA	TGG AGT GGA TGG ATG GGG AT
*mColl10*	AAA CGC CCA CAG GCA TAA AG	CAA CCC TGG CTC TCC TTG G
*mAnk*	TGT CAA CCT CTT CGT GTC CC	GAC AAA ACA GAG CGT CAG CG
*mAnx5*	CCT CAC GAC TCT ACG ATG CC	AGC CTG GAA CAA TGC CTG AG
*mPit1*	CTC TCC GCT GCT TTC TGG TA	AGA GGT TGA TTC CGA TTG TGC
*mPit2*	AAA CGC TAA TGG CTG GGG AA	AAC CAG GAG GCG ACA ATC TT
*mPC1*	CTG GTT TTG TCA GTA TGT GTG CT	CTC ACC GCA CCT GAA TTT GTT
*mAlp*	TTG TGC CAG AGA AAG AGA GAG A	GTT TCA GGG CAT TTT TCA AGG T
*mAdamts4*	GCC CGA GTC CCA TTT CCC GC	GCC ATA ACC GTC AGC AGG TAG CG
*mAdamts5*	GAC AGA CCT ACG ATG CCA CCC AGC	ATG AGC GAG AAC ACT GAC CCC AGG
*mMmp3*	ATA CGA GGG CAC GAG GAG	AGA AGT AGA GAA ACC CAA ATG CT
*mMmp13*	GCA GTT CCA AAG GCT ACA AC	GCT GGG TCA CAC TTC TCT G
*mTimp1*	CCC ACA AGT CCC AGA ACC GCA G	GCA GGC AAG CA AGT GAC GGC
*mTimp3*	TCC TAG ACC CAG TTC CAT ATA CAC TTC	TTG GAC TTC TGC CAA TT CCT T
*mCD11b*	CTC TTC TGG TCA CAG CCC TA	GGG GGA CAG TAG AAA CAG CC
*mCD18*	AGA CAC AAC CAC ACA GCC AG	CCC AGG AAG AAC AGT CCA GC
*mGapdh*	CTC ATG ACC ACA GTC CAT GC	CAC ATT GGG GGT AGG AAC AC
*hCD11b*	ATG TCC AGA AGA GCA CAC GG	AGG GTC TCA CAA GTC TGG GT
*hCD18*	GCT GGT GTT TGC CAC TGA TG	TCG GTG AGT TTC TCG TAG GTC
*hGapdh*	GAT TTG GTC GTA TTG GGC	CTC GCT CCT GGA AGA TGG

### Alkaline Phosphatase Activity Assay

Cells were lysed in 0.01% sodium dodecyl sulfate (SDS) and alkaline phosphatase (Alp) activity was measured by use of p-Nitrophenyl Phosphate assay (Alkaline Phosphatase Assay Kit, Abcam, ab83369). Lysate was read at 405 nm by spectrophotometric measurement (Spectramax M5e plate reader).

### Fluorescence-Activated Cell Staining

Flow cytometry was used to assess CD11b expression on chondrocytes. Cells were harvested from confluent cultures by treatment with a non-enzymatic cell dissociation buffer (5 mM EDTA, 20 mM HEPES in PBS). Cells were re-suspended in fluorescence-activated cell sorting (FACS) buffer (3% fetal calf serum, 5 mM EDTA in PBS) and incubated with PE anti-CD11b (M1/70, eBioscience), FITC anti-CD45 (30-F11, eBioscience) and FITC anti-F4/80 (BM8, eBioscience) for 30 min at 4°C in the dark. For intracellular staining, cells were permeabilized with Cytofix/Cytoperm solution (BD Biosciences) for 20 min and then incubated with biotin anti-Collagen II (2B1.5, Abcam) followed by a streptavidin APC conjugate antibody. Flow cytometric analyses were performed on LSRII cytometer using FACS Diva6 (Becton Dickinson) and FlowJoX software for data processing. The Imaging flow cytometry was carried out with the ImageStream^®^X Mark II Imaging Flow Cytometer (Merck Millipore, Billerica, MA, United States) using INSPIRE software. For data evaluation, IDEAS software version 6.0 was used.

Proliferation rate of WT and CD11b KO chondrocyte were analyzed by FACS after 3 days of culture by using Cell Proliferation Dye eFluor^TM^ 670 (Invitrogen).

### Mice and Induction of Experimental Osteoarthritis

CD11b KO mice (C57BL/6J background) were provided by Prof. Britta Engelhardt (Theodor Kocher Institute, University of Bern). C57BL/6J purchased from Charles River were used as control mice. All animals were kept in a temperature-controlled environment in a ventilated rack with a 12:12-h light:dark cycle. Food and water were given *ad libitum*.

Control wild-type (WT) and CD11b KO female mice were anesthetized at 12 weeks of age and knee joint instability was induced surgically by partial medial meniscectomy (MNX) of the right knee, and the contralateral knee was sham-operated as control (Kamekura et al., 2005). Animals were sacrificed 2 months after surgery. For the aging model, control WT and CD11b KO mice were sacrificed at 37 weeks of age. For all experimental settings, knees dissected and fixed in 10% formaldehyde.

### MicroCT-Scan

Scanning of mice knees was performed with a SkyScan 1076 X-ray μCT scanning system (SkyScan, Belgium), as previously described (Nasi et al., 2016b). New crystal formations were quantified by tissue volume (mm^3^), its bone mineral density (BMD, g/cm^3^) and its crystal content (μg). Tibial subchondral bone was analyzed as means of bone mineral density (BMD, g/cm^3^) and trabecular thickness (Tb.Th, mm).

### Mouse Knee Histology and Immunohistochemistry

Fixed knees were decalcified in 5% formic acid, dehydrated, and embedded in paraffin. Frontal sections of 6 μm at different deepness of cartilage were stained with Safranin-O-fast green. OARSI scoring of medial tibial plateau and medial femoral condyle was performed (score from 0 to 6 for each, in which 0 represents a healthy cartilage; 0.5 is some loss of safranin-O staining, meaning loss of proteoglycans; 1 some fibrillations in the superficial layer and no loss of cartilage; 2 erosion of the surface; 3 to 5 represent various percentages of the deepness of the cartilage affected by vertical clefts and erosion) (Glasson et al., 2010). Furthermore, inflammation of the synovial membrane was scored (each deepness of the sections was scored on a 0 to 3 scale of inflammation, then the average was calculated for one sample). Paraffin-embedded knee sections were stained with CD11b antibody (133357, Abcam). MMP13 was detected with anti-MMP13 (Ab39012 from Abcam), and quantified by calculating positive cells by use of Image J (U. S. National Institutes of Health, Bethesda, MD, United States).

### Human Cartilage Explants Preparation and Chondrocytes Isolation

Cartilage explants were obtained from 13 human OA patients undergoing total knee replacement surgery from Lausanne University Hospital, Switzerland (average age 71 years old ± 11). Sections of macroscopically undamaged and damaged cartilage were dissected from the femoral condyles and processed for histological analysis from 3 patients. The rest of undamaged and damaged cartilage was cut in small pieces, washed twice with PBS and twice with DMEM. Cartilage was degraded for 10 h in 10% Liberase (Roche) DMEM, and digested tissue passed through a 70 μm filter (BD biosciences) to obtain articular chondrocytes. Cells were plated and cultured as described above.

### Statistical Analysis

All values are expressed as the mean ± SD. Variation between data sets was evaluated using the Student’s *t*-test or one-way or two-way ANOVA test, where appropriate. Differences were considered statistically significant for a value of *p* < 0.05 (^∗^*p* < 0.05, ^∗∗^*p* < 0.01, ^∗∗∗^*p* < 0.001, ^****^p < 0.0001). Data was analyzed with GraphPad Prism software (GraphPad software), San Diego, CA.

### Ethics Statement

Experiments on mice were performed in strict accordance to the Swiss Federal Regulations. The protocol was approved by the “*Service de la consommation et des affaires vétérinaires du Canton de Vaud*,” Switzerland. All efforts were made to minimize suffering and minimize the number of mice needed to assess statistical significance and experimental reproducibility. Human samples were obtained with the approval of the Centre Hospitalier Universitaire Vaudois ethical committee and patients’ written informed consent.

## Results

### CD11b Integrin Is Expressed in Murine and Human Articular Cartilage

**FIGURE 1:**
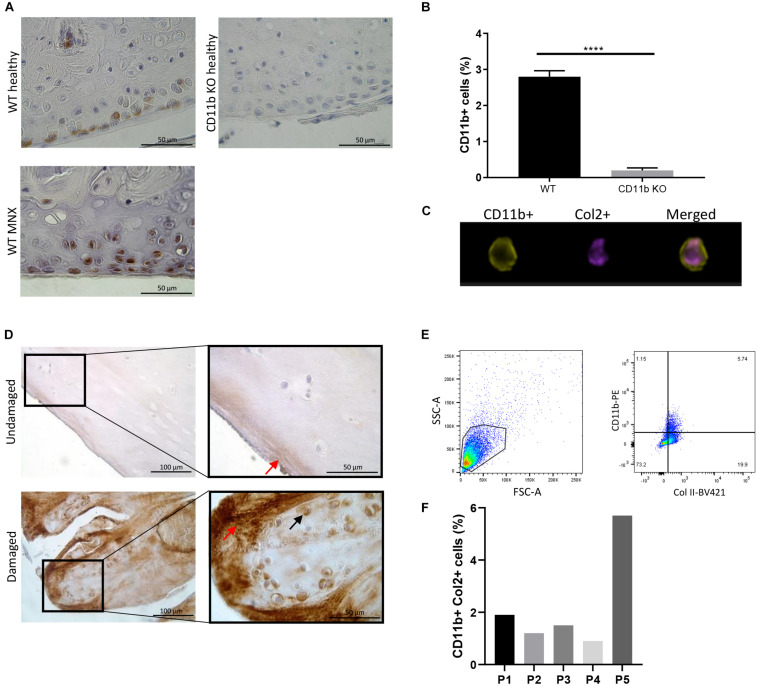
CD11b integrin is expressed in murine and human articular cartilage. **(A)** CD11b immunohistochemical staining (brown) in knee cartilage from healthy WT and CD11b KO mice, and meniscectomized (MNX) WT mice. For each group, one representative picture from one out of 3 mice is shown. Scale bars 50 μm. **(B)** FACS analysis and quantification of CD11b positive and Col2 positive primary murine chondrocytes, and **(C)** Amnis FACS analysis of CD11b positive (green) and Col2 positive (purple) staining; ****p < 0.0001. **(D)** CD11b immunohistochemical staining (brown) in undamaged and damaged femoral knee cartilage from OA patients undergoing joint replacement (black arrows indicate intracellular CD11b expression, red arrows indicate extracellular CD11b expression). For each group, one representative picture from one (P13) out of 3 patients is shown. Scale bars 100 and 50 μm. **(E,F)** Flow cytometry analysis of CD11b and Col2 expression on primary human chondrocytes obtained from cartilage of 5 osteoarthritis patients.

To determine the distribution of CD11b expression in the mouse cartilage, CD11b immunostaining was performed on knee sections from WT and CD11b KO mice. As seen in Figure 1A (left panels), positive signal was primarily detected in chondrocytes in the superficial layer of cartilage both in healthy and meniscectomized WT knee joints (MNX). Verifying specificity of the antibody, no CD11b staining was detected in articular chondrocytes of CD11b KO mice (Figure 1A, top right panel).

This data was confirmed by flow cytometry analysis. Indeed, 3–4% of WT primary chondrocytes extracted from articular cartilage expressed the CD11b integrin subunit (Figure 1B and Supplementary Figure 1D). Combining high-resolution microscopy and FACS (Amnis analysis), we found that CD11b positive cells also expressed collagen II, a specific marker of chondrocytes (Figure 1C). In addition, the chondrocytes showed no expression of F4/80, a macrophage marker (Supplementary Figure 1D) and were negative for CD45 (Supplementary Figure 1E), an antigen used to define leukocyte populations. Furthermore, CD11b expression in murine chondrocytes was demonstrated by q-PCR (Supplementary Figure 1A).

We next analyzed if CD11b was expressed in human cartilage by CD11b immunostaining analysis. Cartilage was obtained from the knee joint of three OA patients and separated into macroscopically healthy and damaged cartilages. The results in Figure 1D and Supplementary Figure 1C showed that CD11b was present in chondrocytes in the superficial layer of human articular cartilage (black arrows). Interestingly, a diffuse expression pattern of CD11b at the extracellular matrix was also observed in the damaged cartilage (red arrows). In Figures 1E,F, CD11b expression in human chondrocytes was confirmed by flow cytometry analysis, with an expression level varying from 2 to 6% of the cells. Further proof of CD11b expression by human chondrocytes was obtained by qPCR (Supplementary Figure 1B), showing both CD11b and CD18 expression, with a predominant expression of CD18. We hypothesized that extracellular matrix deposited CD11b could result from shedding of the integrin (Gomez et al., 2012). Indeed, we detected by ELISA measurement soluble CD11b in human OA synovial fluid (24 patients, average 4.5 ng/ml ± 0.4).

### CD11b Deficiency Increases Mineralization by Murine Joint Chondrocytes

**FIGURE 2:**
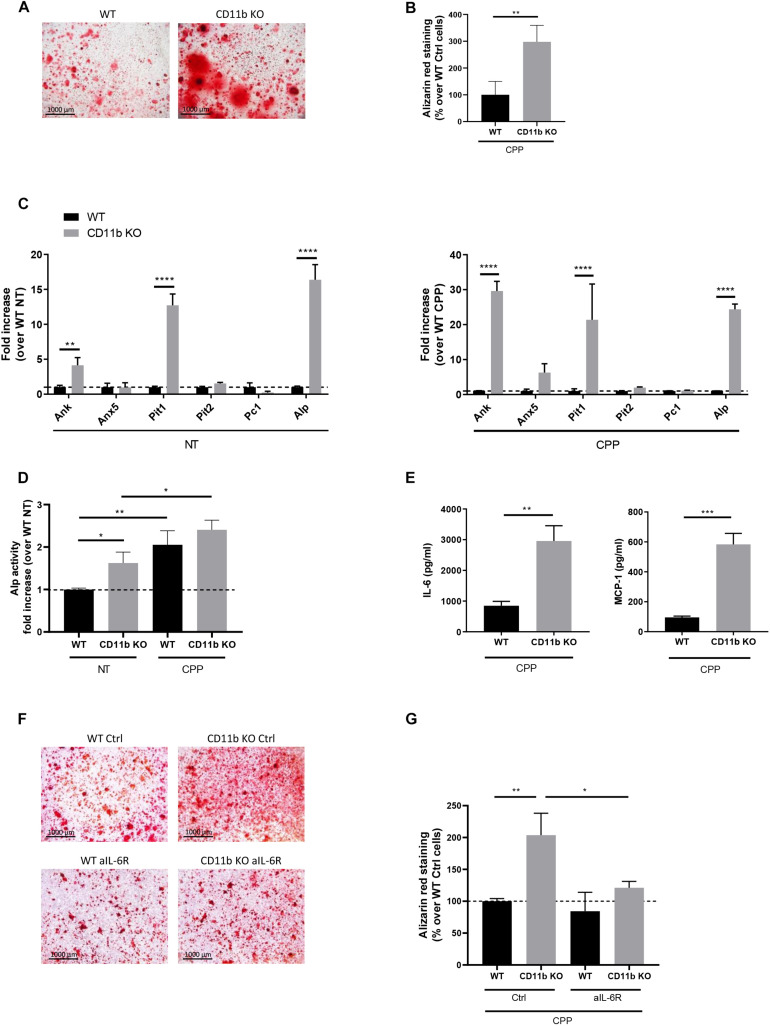
CD11b deficiency increases mineralization by murine articular chondrocytes. **(A)** Alizarin Red staining of WT and CD11b KO primary murine chondrocytes stimulated with secondary CPP for 24 h in DMEM + 10% FBS. Pictures show one representative culture well of one experiment out of three independent experiments. Scale bars 1000 μm. **(B)** The graph shows the percentage of Alizarin Red positive surface over total surface, calculated in Adobe^®^ Photoshop^®^ and normalized on WT chondrocytes. Values represent means ± SD of triplicates samples from one representative experiment out of three independent experiment; **p < 0.01. **(C)** qRT-PCR of the indicated genes in primary murine chondrocytes stimulated or not with secondary CPP for 4 h. Values represent fold increase of CD11b KO chondrocytes over their WT control cells (dotted line), shown in means ± SD of triplicates samples; **p < 0.01, ****p < 0.0001. **(D)** Quantification of Alkaline phosphatase activity in WT and CD11b KO primary murine chondrocytes treated or not with secondary CPP for 6 h. Values represent fold increase of Alp activity in CD11b KO over WT NT cells (dotted line) as means ± SD of triplicates samples; *p < 0.05, **p < 0.01. **(E)** IL-6 and MCP-1 concentration in cell supernatants of WT and CD11b KO primary murine chondrocytes of point (A). Values represent means ± SD of triplicates samples; **p < 0.01, ***p < 0.001. **(F)** Alizarin Red staining of WT and CD11b KO murine chondrocytes stimulated with secondary CPP for 24 h in DMEM + 10% FBS and treated or not with an anti-IL-6 receptor (aIL-6R, 10 μg/ml) or its IgG control (Ctrl, 10 μg/ml). Scale bars 1000 μm. **(G)** The graph shows the percentage of Alizarin Red positive surface over the total surface, calculated in Adobe^®^ Photoshop^®^ and normalized on control WT chondrocytes. Values represent means ± SD of triplicates samples; *p < 0.05, **p < 0.01.

We then evaluated the effect of CD11b deficiency on chondrocyte mineralization. Primary murine chondrocytes were allowed to mineralize in the presence of calciprotein particles (CPP) and mineralization was assessed after 24 h by Alizarin Red. As shown in Figure 2A, CD11b KO chondrocyte culture exhibited massive calcium containing crystal deposits compared to WT control (in red). Quantification of Alizarin Red staining showed a 3-fold significant increase of crystals in CD11b KO cells (Figure 2B). The increased calcifying ability of CD11b KO chondrocytes was not restricted to the model used, as we obtained similar results with another model of calcification (complete medium with 50 μg/mL ascorbic acid and 20 mM β-glycerol phosphate for 14 days), *i.e.*, CD11b KO chondrocytes mineralized more compared to WT control (Supplementary Figure 2A). This effect was not due to cytotoxicity, as measured by lactate dehydrogenase (LDH) activity (Supplementary Figure 2B).

To characterize how CD11b might interfere with calcification in chondrocyte cultures, we assessed changes in the expression of genes involved in the calcification process (Ea et al., 2011) (*Ank, Anx5*, *Pit1*, *Pit2*, *Pc1* and *Alp*) by qRT-PCR. Results in Figure 2C show that *Ank*, *Pit1* and *Alp* expression were significantly increased in CD11b KO chondrocytes both in unstimulated or calcifying conditions (CPP). Regarding *Pit2* and *Pc1*, no significant differences were observed. We provided further confirmation of the calcifying phenotype of CD11b KO cells by measuring the activity of alkaline phosphatase (Alp) in the cell lysate in non-treated and CPP treated chondrocytes. The Alp specific activity was higher in CD11b KO cells compared to that of WT control (Figure 2D). Altogether, these results demonstrate that CD11b deficiency in chondrocytes enhanced the expression of calcification genes.

### CD11b Deficiency Increases Secretion of the Pro-mineralizing IL-6 Cytokine by Murine Chondrocytes

We previously demonstrated that IL-6 had pro-mineralizing effects in chondrocytes and, reciprocally, BCP crystals increased IL-6, thus leading to a vicious circle (Nasi et al., 2016b). Hence, we speculated that increased IL-6 secretion by CD11b-deficient chondrocytes could favor mineralization. Indeed, we detected by ELISA increased IL-6 levels in the medium from CD11b KO chondrocytes stimulated with CPP (Figure 2E). Additionally, CPP stimulation significantly increased MCP-1 secretion in CD11b deficient chondrocytes compared to WT control. Next, to understand if IL-6 secretion by CD11b KO chondrocytes was the main calcification inducer in these cells, we added anti IL-6 receptor antibody and CPP on WT and CD11b KO chondrocytes (Figure 2F). Indeed, by blocking IL-6 signaling in CD11b KO cells, the effect on calcification was abrogated and restored to WT levels, as confirmed by quantification in Figure 2G. Therefore, deficiency of CD11b in chondrocytes enhances the pro-inflammatory molecules IL-6 and MCP-1, sustaining the loop between inflammation and calcification.

### CD11b in Chondrocytes Regulates Genes Involved in Hypertrophy and Extracellular Matrix Degradation

**FIGURE 3:**
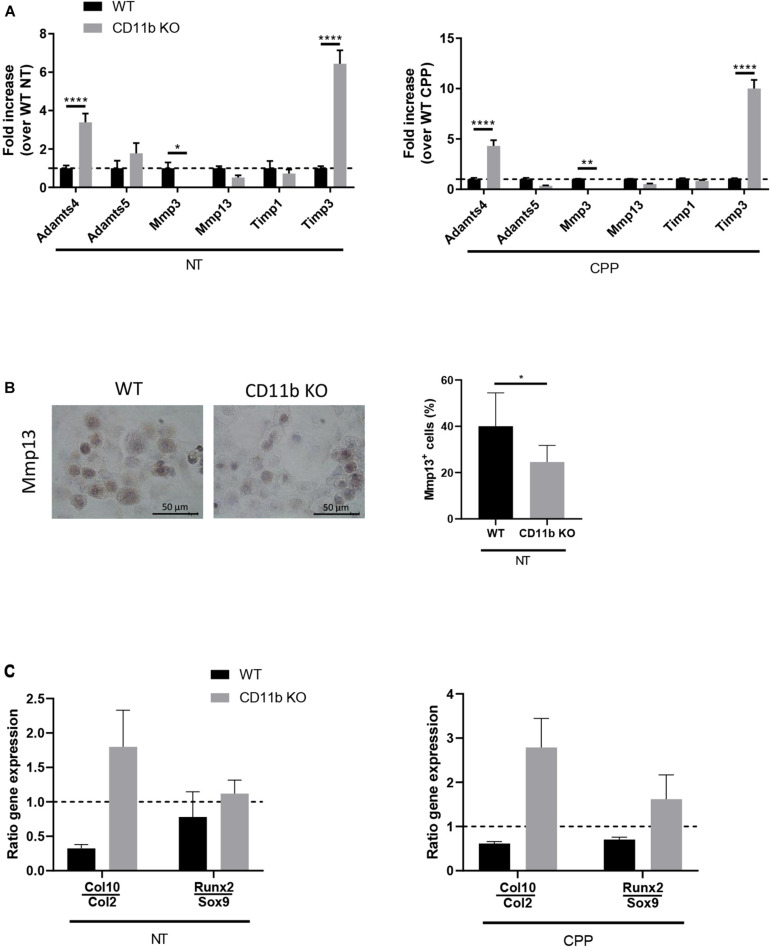
CD11b deficiency in chondrocytes alters the expression of matrix-degrading enzymes and is associated with a pro-hypertrophic expression profile. **(A)** qRT-PCR of the indicated genes in WT and CD11b KO primary murine chondrocytes at basal level (left graph) or in cell stimulated with CPP for 4 h (right graph). Values represent fold increase of CD11b KO chondrocytes over their WT control cells, shown in means ± SD of triplicates samples; *p < 0.05, **p < 0.01, ****p < 0.0001. **(B)** Mmp13 immunohistochemical staining (brown) in primary murine chondrocytes from WT and CD11b mice. For each group, one representative picture out of triplicates wells is shown. Scale bars 50 μm. The graph shows quantification of Mmp13 expression calculated as the percentage of positive cells in each field. Three fields were scored for each triplicate wells and means ± SD plotted; *p < 0.05. **(C)** qRT-PCR of the indicated genes in WT and CD11b KO primary murine chondrocytes at basal level (left graph) or in cells stimulated with CPP for 4 h (right graph). Values represent the ratio between late-stage hypertrophic differentiation markers over early-stage differentiation genes.

In order to investigate the mechanisms underlying the effects of CD11b integrin on extracellular matrix degradation *in vitro*, we analyzed the gene expression level of key catabolic factors in WT and CD11b KO chondrocytes after 4 h exposure to CPP and their non-treated controls. We focused our analysis on metalloproteases (*Adamts4*, *Adamts5*, *Mmp3*, *Mmp13*) and inhibitors of metalloproteases (*Timp1*, *Timp3*), whose expression plays a major role in chondrocyte extracellular matrix degradation during OA (Tseng et al., 2009; Troeberg and Nagase, 2012; Roughley and Mort, 2014). As shown in Figure 3A, CD11b KO chondrocytes, with or without stimulation with CPP, showed significantly higher levels of *Adamts4* and *Timp3* mRNA than in control WT chondrocytes. While *Timp1, Adamts5*, and *Mmp13* mRNA had no significant changes, there was lower *Mmp3* in CD11b KO chondrocytes.

Following expression level analysis, we investigated the presence of Mmp13 by immunohistochemistry (Figure 3B, left). Mmp13 was significantly lowered in CD11b deficient chondrocytes, as confirmed by Image J quantification (Figure 3B, graph on the right).

We then analyzed, by qPCR, markers of articular cartilage hypertrophy including early differentiation *Sox9* and *Col2*, and hypertrophy markers *Runx2* and *Col10* (Figure 3C). The ratio calculated on *Col10* over *Col2* and *Runx2* over *Sox9* revealed an increase in hypertrophic phenotype in CD11b KO chondrocytes, both at basal level and in CPP treated condition. Finally we ruled out that chondrocyte proliferation was affected by CD11b deficiency, as the proliferation response of isolated WT and CD11b KO chondrocytes by FACS using dye dilution assays for cell proliferation was similar (Supplementary Figure 2C).

### Targeting CD11b With LA1 Agonist Results in Reduced Murine and Human Chondrocytes Calcification *in vitro*

**FIGURE 4:**
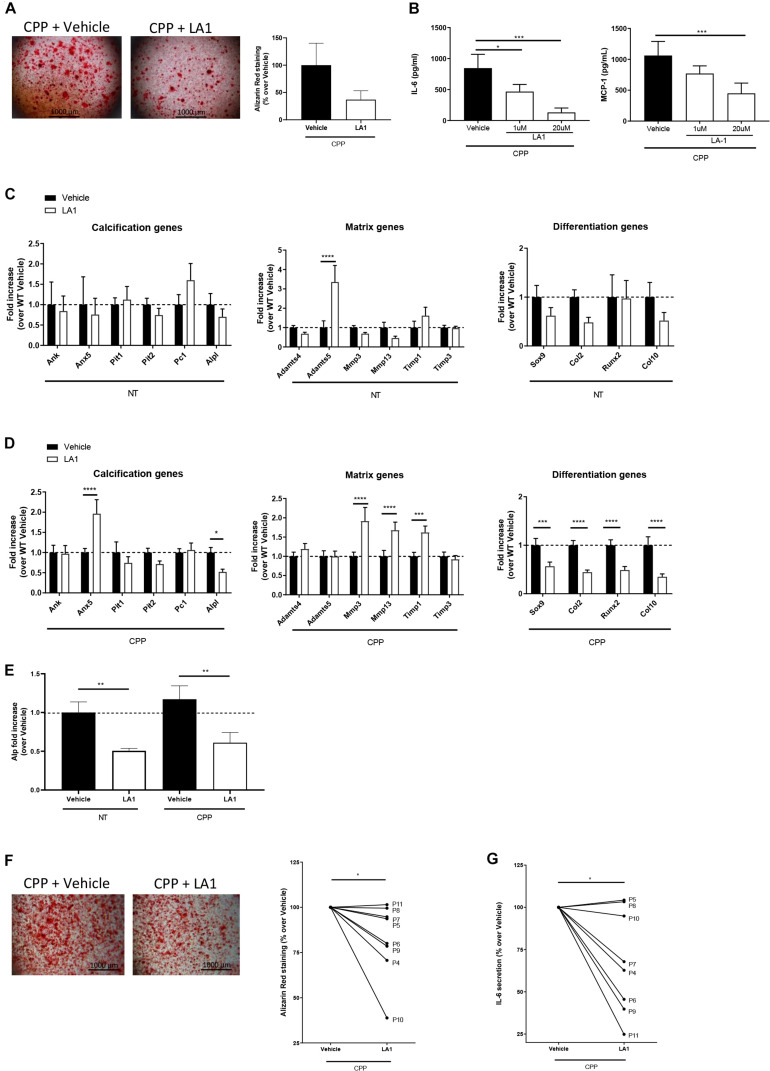
CD11b agonist reduces murine and human chondrocytes calcification *in vitro.* **(A)** Alizarin Red staining of WT primary murine chondrocytes stimulated with secondary CPP ± LA1 (20 μM) for 24 h in DMEM + 10% FBS. Pictures show one representative culture well of one experiment out of three independent experiments. The graph shows the percentage of Alizarin Red positive surface over the total surface, normalized on Vehicle treated cells. Values represent means ± SD of triplicates samples. **(B)** IL-6 and MCP-1 concentration in cell supernatants of WT primary chondrocytes stimulated with secondary CPP and LA1 at different concentration (0 μM - Vehicle-, 1 μM and 20 μM). Values represent means ± SD of triplicates samples; *p < 0.05, ***p < 0.001. **(C)** qRT-PCR of the indicated genes in murine chondrocytes stimulated with LA1 (20 μM) or its vehicle for 4 h. Values represent fold increase of LA1 treated chondrocytes over their Vehicle-treated cells, shown in means ± SD of triplicates samples; ****p < 0.0001. **(D)** qRT-PCR of the indicated genes in WT primary murine chondrocytes stimulated with secondary CPP ± LA1 (20 μM) for 4 h. Values represent fold increase in LA1 treated chondrocytes over Vehicle-treated cells, shown as means ± SD of triplicates samples; *p < 0.05, ***p < 0.001, ****p < 0.0001. **(E)** Quantification of Alkaline phosphatase activity in WT chondrocytes treated with secondary CPP ± LA1 for 6 h. Values represent fold increase of Alp activity in LA1 treated chondrocytes over Vehicle-treated cells treated, shown as means ± SD of triplicates samples; **p < 0.01. **(F)** Alizarin Red staining of human primary chondrocytes stimulated with secondary CPP and treated with LA1 or its Vehicle. Pictures show one representative culture well of one OA patient (P9) out of eight patients. The graph shows the corresponding Alizarin Red positive surface over the total surface, calculated in Adobe^®^ Photoshop^®^, and then normalized. Lines connect the two conditions for each patient, values are calculated over CPP-Vehicle and represent means of triplicates samples; *p < 0.05. **(G)** IL-6 concentration in supernatants of human primary chondrocytes at point **(F)**. Values represent means of triplicates samples; *p < 0.05.

It has been reported that treatment with CD11b agonist LA1 led to integrin activation and suppression of inflammation in macrophages (Faridi et al., 2017). Given the relationship between inflammation and chondrocyte calcification, we hypothesized that CD11b activation may play a protective role during the process of calcification. To assess the hypothesis, primary WT murine chondrocytes were allowed to calcify for 24 h with CPP in the presence or absence of LA1, and assessed by Alizarin Red staining. We found that LA1 treatment significantly reduced chondrocyte mineralization (Figure 4A), without any associated *in vitro* cytotoxicity as measured by LDH activity (Supplementary Figure 3A). Importantly, we also found that LA1 significantly inhibited IL-6 and MCP-1 secretion in a dose dependent manner confirming the anti-inflammatory role of LA1-mediated integrin activation in chondrocytes (Figure 4B).

To further demonstrate that CD11b activation on chondrocytes prevents cartilage calcification *in vitro*, we performed RT-qPCR analysis of the previously investigated genes related to calcification, differentiation and extracellular matrix turn-over. In Figure 4D, *Anx5* was significantly increased in WT chondrocytes treated with LA1, compared to control, while *Alp* was decreased. This latter modulation is in line with the previous result in which CD11b KO cells had increased *Alp*. The genes *Sox9*, *Col2*, *Runx2*, *Col10*, *Mmp3*, *Mmp13* and *Timp1* were all decreased with LA1 stimulation. In LA1 treated chondrocytes without CPP, *Adamts5* was significantly increased compared to control cells (Figure 4C). Furthermore, in Figure 4E, we investigated Alp activity in WT chondrocytes treated with LA1 or its control, and found that LA1 decreased Alp both with CPP and without.

To further evaluate the anti-calcifying effect of LA1 on human cells, we performed Alizarin Red staining on human primary chondrocytes extracted from damaged cartilage (8 OA patients). LA1 reduced mineralization in six out of eight primary chondrocyte cultures (Figure 4F). Moreover, in the same conditions LA1 significantly reduced IL-6 levels in the supernatant (Figure 4G). Finally, the anti-calcifying property of LA1 was not restricted to chondrocytes, as LA1 was also able to inhibit crystal deposition in human primary synoviocytes purified from OA patient’s synovial membrane (Supplementary Figure 3B), after calcification induction.

### Cartilage Damage and Periarticular Crystal Deposits Are Exacerbated in CD11b Deficient Mice Following Experimental OA

Based on the increased propensity of CD11b-deficient chondrocytes to mineralize and to produce IL-6 and on the increased levels of IL-6 produced *in vivo* (Stevanin et al., 2017), we hypothesized genetic ablation of CD11b in mice would predispose these animals to OA.

**FIGURE 5:**
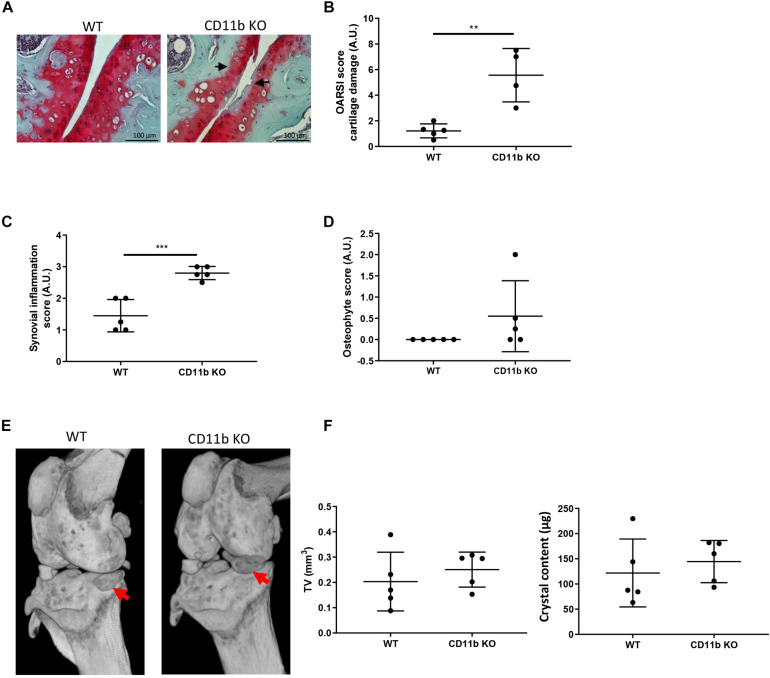
Cartilage damage and periarticular crystal deposits are exacerbated in CD11b deficient mice following experimental OA. **(A)** Representative Safranin-O histological staining of knee joints from WT and CD11b KO mice 8 weeks after MNX. Black arrows show degenerative OA changes in CD11b KO mice. Scale bars 100 μm. **(B)** Corresponding total OARSI score for cartilage damage in medial compartment of WT and CD11b KO knees. Values represent means ± SD; **p < 0.01. **(C)** Synovial inflammation scoring in WT and CD11b KO MNX mice knees; ***p < 0.001. **(D)** Osteophyte scoring in WT and CD11b KO MNX mice knees. **(E)** Representative micro-CT scan images of murine knee joints from WT and CD11b KO mice of point (A). Red arrows show calcified deposits in meniscectomized knees. **(F)** Corresponding CT Analyzer quantitative analysis of new formation volume (TV: tissue volume, mm^3^) and new formation crystal content (μg) in WT and CD11b KO menisectomized mice. Data are expressed as the mean ± SD.

We analyzed the development of instability-induced OA changes in CD11b deficient and WT mice using the knee meniscectomy (MNX) model as previously described (Nasi et al., 2016b), and sacrifice after 8 weeks from the knee surgery. Cartilage damage, as evidenced by OARSI scoring of medial tibial plateau and medial femoral condyle, was significantly increased in CD11b-deficient mice, with decreased proteoglycans content (less Safranin-O staining), more fissurations and fibrillations compared to WT control mice (Figures 5A,B). Moreover, the joints of CD11b KO mice exhibited significantly increased synovial inflammation (Figure 5C), and increased, but not significant, osteophyte formation (Figure 5D) in CD11b KO mice, with respect to their WT control.

In addition, microCT-scan examination was conducted to analyze the crystal deposition (Figure 5E). By CT Analyzer quantification, both the volume of newly formed crystals as well as the overall crystal content, were slightly increased in CD11b KO mice compared to WT control, although these differences did not reach statistical significance (Figure 5F).

In order to rule out an underlying cartilage or bone phenotype in CD11b KO mice, that may predispose them to OA, we analyzed cartilage damage and subchondral bone structure in unchallenged WT and CD11b KO mice. In the first experimental setting we considered knees from sham-operated 20 weeks old WT and CD11b KO mice. OARSI scoring revealed no difference in cartilage degradation between WT and CD11b KO mice (Supplementary Figures 4A,B). Moreover, no difference was seen in subchondral bone mineral density and subchondral bone trabecular thickness (Supplementary Figures 4C,D) between the two different genotypes. Similar results were obtained in aged WT and CD11b KO mice (37 weeks old), where no difference in cartilage damage (Supplementary Figure 4E,F) nor in subchondral bone parameters was seen (Supplementary Figures 4G,H).

## Discussion

Integrin CD11b/CD18 is a receptor originally described on neutrophils and macrophages, known to support adhesion and molecular cross-talk of these cells with the extracellular matrix proteins. It regulates many functions including phagocytosis, migration and immune tolerance (Ehirchiou et al., 2007; Rosetti and Mayadas, 2016), but it can also bind to a great number of different ligands, among which fibrinogen (Rosetti and Mayadas, 2016), fibronectin and laminin (Bohnsack and Zhou, 1992) can also be found in cartilage. CD11b/CD18 is generally thought to be exclusively expressed on leukocytes. In this work, we showed that CD11b is also expressed on chondrocytes, and importantly, established a novel role of CD11b integrin signaling in cartilage calcification and degradation in a mouse model of OA.

We first demonstrated the expression of the CD11b integrin subunit on primary murine and human chondrocytes and in articular cartilage of both murine and human OA patients by combining flow cytometry, qRT-PCR analysis and immunohistochemistry. CD11b is expressed only on a subset of chondrocytes in the superficial zone of the articular cartilage, and by FACS we confirmed 3–4% of chondrocytes expressing this integrin subunit. In line, in an open access database of single cell RNA sequencing on human OA chondrocytes (Ji et al., 2019), the gene encoding for CD11b was expressed in 3.15% of cells (analysis not shown), and these CD11b expressing cells also expressed Col2 as a marker of chondrocytes. Our results are also in agreement with a previous study reporting a very low expression of β2 integrin on chondrocytes (Iannone et al., 2001). Surprisingly, we also found CD11b in the extracellular matrix of cartilage from OA patients. This could be accounted for by cleavage of the extracellular domain of CD11b from the cell surface after cell activation under inflammatory conditions. This cleavage involves matrix metalloproteases and leads to subsequent binding of cleaved CD11b to extracellular matrix proteins (Zen et al., 2011). The presence of soluble complexes of CD11 and CD18 have been reported in human plasma and in synovial fluid from patients with arthritis (Gjelstrup et al., 2010). We confirmed the presence of soluble CD11b in the synovial fluid of OA patients that we analyzed, although cellular sources and targets of soluble CD11b remain to be determined. In OA joints, we postulate that the main source could be synovial cells and infiltrating inflammatory cells such as macrophages and neutrophils. Indeed, these cells express high levels of CD11b and inflammation-induced matrix metalloproteases which could contribute to CD11b shedding (Gjelstrup et al., 2010; Manferdini et al., 2016). The pathological relevance of extracellular CD11b is unclear and further investigations will be needed to determine the contribution of the truncated form of CD11b on OA progression.

We demonstrated that CD11b KO chondrocytes mineralized more than WT chondrocytes and that this could be via multiple mechanisms. First, CD11b KO chondrocytes showed more active calcification machinery compared to WT cells. Calcium-containing crystals, and in particular BCP crystals, are formed when excess of calcium (Ca^2+^) and inorganic phosphate (P_*i*_) are uptaken into matrix vesicles (Anderson et al., 2004; Ea et al., 2011). Crystal formation starts when inorganic pyrophosphate (PP_*i*_) is extruded extracellularly via the multipass transmembrane transporter Ank. Extracellular PP_*i*_ is then hydrolyzed to P_*i*_ by the enzyme tissue-non-specific alkaline phosphatases (Alp). Finally, P_*i*_ is concentrated in matrix vesicles through the cotransporters Pit1/Pit2 and Ca^2+^ through annexin 5 (Anx5). Here we demonstrated that CD11b KO chondrocytes, both at basal- and CPP-condition, express higher levels of the pro-mineralizing genes *Ank, Alp* and *Pit1*, and have more active Alp enzyme. Down-regulation of these calcification factors could be a potential mechanism by which CD11b exerts its anti-mineralizing properties in chondrocytes. From the literature, we can speculate possible cross-talks between CD11b-dependent signaling pathways such as JNK and NF-kB and these calcification players. Activation of the JNK (Suzuki et al., 2006; Wu et al., 2016) or of the NF-kB (Koumakis et al., 2019) pathway has been demonstrated to induce *Pit1* expression and subsequent cell matrix mineralization or inflammation respectively. JNK and NF-kB activation were also associated to increased *Alp* expression and osteoblastic differentiation of arterial smooth muscle cells (Chang et al., 2020). As a second calcification mechanism, in our study we found that CD11b deficient chondrocytes secreted increased amounts of pro-inflammatory and pro-mineralizing IL-6 and MCP-1 when stimulated with CPP. More importantly, by blocking the receptor of IL-6 on CD11b KO chondrocytes we were able to greatly decrease calcification. Another characteristic of mineralizing chondrocytes is hypertrophy. Indeed, CD11b deficiency induced considerable upregulation of Col10 and downregulation of Col2, as shown by the ratio, indicating that CD11b deficient chondrocytes are in a hypertrophic state. In summary, we demonstrated that CD11b is an important negative regulator of mineralization in chondrocytes and most if not all of its anti-mineralizing effects could be accounted by decreased pro-mineralizing IL-6 cytokine levels (Nasi et al., 2016b). To address the underlying mechanisms involved in enhanced cartilage degradation of CD11b-deficient mice after MNX, we analyzed the key ECM markers under this disease setting. Surprisingly, deletion of the CD11b gene did not cause a coordinated increased expression of enzymes related to ECM degradation, as the pro-catabolic aggrecanase *Adamts4* was upregulated in CD11b KO chondrocytes concomitantly with its inhibitor *Timp3*. Another pro-catabolic metalloprotease *Mmp1*3 was downregulated in CD11b KO chondrocytes. Upon stimulation with the CD11b agonist LA1, both *Adamts5* and its inhibitor *Timp1* were increased. Given these expression patterns of ECM enzymes, we ruled out their early modulation as a crucial mechanism explaining the more severe phenotype observed in CD11b KO cells and mice, and the *in vitro* protective effect of LA1.

However, as mentioned above, measurement of the supernatant from stimulated CD11b KO chondrocytes showed an increase in both IL-6 and MCP-1 levels, which are known players in cartilage catabolism, alongside cartilage calcification (Kapoor et al., 2011; Nasi et al., 2016b).

We found that increased cartilage degradation is associated with synovial inflammation and the formation of osteophytes in CD11b KO mice after meniscectomy, while we could rule out that an underlying pre-existing pathology or bone phenotype exists in sham-operated knees and knees of old CD11b KO mice, which may predispose them to OA. Our data suggest that modulating CD11b could be an effective strategy to protect the cartilage in experimental OA. A growing body of literature demonstrates the role of Leukadherin-1 (LA1) mediated activation of CD11b in regulating inflammation in experimental models of inflammatory disorders. By binding to an allosteric pocket in the CD11b subunit, LA1 changes the conformation of the extracellular domain, thereby preventing infiltration of pro-inflammatory monocytes after organ injury and vascular dysfunction (Maiguel et al., 2011; Schmid et al., 2018). In a study by C. Han et al. (2010), the authors show that CD11b, once activated by Toll-Like Receptor (TLR), triggers spleen tyrosine kinase (Syk) activation and phosphorylation of MyD88 and TRIF, which are then degraded by proteolysis, thereby reducing IL-6, TNF and IFN-β downstream production. Another study (Faridi et al., 2017) demonstrated that LA1 activated CD11b which in turn inhibited MyD88 and TRIF downstream pathway. The effects of LA1 on chondrocytes have not been studied. Our results demonstrated the anti-inflammatory effect of LA1 in chondrocytes. Indeed, we found that WT chondrocytes cultured with LA1 showed a reduction in calcification and a decrease in production of the IL-6 and MCP-1. Additionally, we also obtained significant reduction in calcification and IL-6 release in human chondrocytes treated with LA1. Finally, as human OA synoviocytes can also calcify (Sun et al., 2014), we showed that LA1 can decrease mineralization of these cells, thereby confirming a general anti-mineralizing effect of LA1. On the other hand, we ruled out that the anti-mineralizing effect of LA1 was mediated by reduced chondrocyte hypertrophy, as LA1 suppressed the expression of both early-stage (Coll2, Sox9) and late-stage hypertrophy (Coll10, Runx2) chondrocitic markers.

However, we cannot rule out the possibility that LA1 can block calcification via a CD11b-independent mechanism, which deserves further investigation.

While a direct association between CD11b and calcification has not been reported, genome-wide association studies have established a strong correlation between single-nucleotide polymorphisms (SNPs) in the *ITGAM* gene coding for CD11b and susceptibility to SLE (Hom et al., 2008; Nath et al., 2008). These SNPs produce a dysfunctional CD11b protein on leukocytes that is deficient in many functions including activation, ligand binding, cell adhesion and phagocytosis (Khan et al., 2018). Moreover, Dahdal et al. reported that SLE patients have significantly elevated calcification propensity in their serum (Dahdal et al., 2018). In addition, earlier research has shown a link between coronary artery calcifications and increased disease activity in young lupus patients (Romero-Diaz et al., 2012). Finally, an association between IL-6 and progression of lupus has been demonstrated using several murine models of SLE (Tang et al., 1991; Alarcon-Riquelme et al., 1992; Kobayashi et al., 1992). However, it is still unknown how the SNPs contribute to the calcification propensity seen in lupus patients. Our data suggest a strong direct link between reduced CD11b function and increased calcification, which can be explained by the inability of CD11b KO chondrocytes to downregulate the pro-inflammatory cytokine and chemokine production.

Although the contribution of CD11b integrin in calcification is clearly established in the present study, we did not investigate which ligands bind to CD11b in the cartilage. Integrin CD11b is a multi-ligand receptor capable of binding ICAM-1, fibrinogen, fibronectin, factor X and complement factor iC3b, among many others (Rosetti and Mayadas, 2016). Integrin ligands have been shown to play a crucial role in OA (Pfander et al., 1999; Pullig et al., 2000). Indeed, previous studies have demonstrated that following cartilage damage, fragmented extracellular matrix molecules can trigger catabolic gene expression in various cells in the joint tissues (Homandberg et al., 2002; Loeser et al., 2003). Further studies will be required to investigate the contribution of CD11b ligands in OA cartilage.

Even though our current work unveils a new pathway in OA, it also presents some limitations that have to be considered. The use of global CD11b KO mice does not allow us to determine whether the observed effects are solely due to lack of CD11b expression in chondrocytes and not due to other cells in the surrounding tissues of the joint. This question will be resolved in future by generating mice with a selective CD11b deficiency in cartilage.

In summary, our study showed for the first time that CD11b signaling plays an important role in regulating cartilage calcification and OA. Deletion of CD11b gene was linked with cartilage degradation in knee joints during OA, mediated by upregulation of the pro-inflammatory IL-6 and MCP-1. Moreover, activation of CD11b with the LA1 agonist decreased the levels of pro-inflammatory cytokines and prevented calcification *in vitro*. This study offers novel insights into the role of CD11b integrin signaling in regulatory mechanisms that control OA severity and progression, and provides a new target for future therapies, with final aim of improving the quality of life in OA patients.

## Data Availability Statement

The raw data supporting the conclusions of this article will be made available by the authors, without undue reservation.

## Ethics Statement

The studies involving human participants were reviewed and approved by Centre Hospitalier Universitaire Vaudois ethical committee, Lausanne, Switzerland. The patients/participants provided their written informed consent to participate in this study. The animal study was reviewed and approved by “Service de la Consommation et des Affaires Vétérinaires du canton de Vaud,” Switzerland.

## Author Contributions

DE and IB designed, performed and analyzed experiments, and helped with the preparation of the manuscript. VC and MC performed experiments. TH, AS, and LZ provided advice and help with the preparation of the manuscript. NB conceived, supervised the study, and wrote the manuscript. SN performed some experiments, supervised the study and wrote the manuscript. All authors contributed to the article and approved the submitted version.

## Conflict of Interest

The authors declare that the research was conducted in the absence of any commercial or financial relationships that could be construed as a potential conflict of interest.
