# Supplementary material for: CD11b Signaling Prevents Chondrocyte Mineralization and Attenuates the Severity of Osteoarthritis
Source: Front Cell Dev Biol. 2020 Dec 18;8:611757. doi: 10.3389/fcell.2020.611757 (PMC7775404; doi:10.3389/fcell.2020.611757)
Supplement: Supplementary file 1 [file Data_Sheet_1.docx]

Supplementary Material

# Supplementary Figures

##
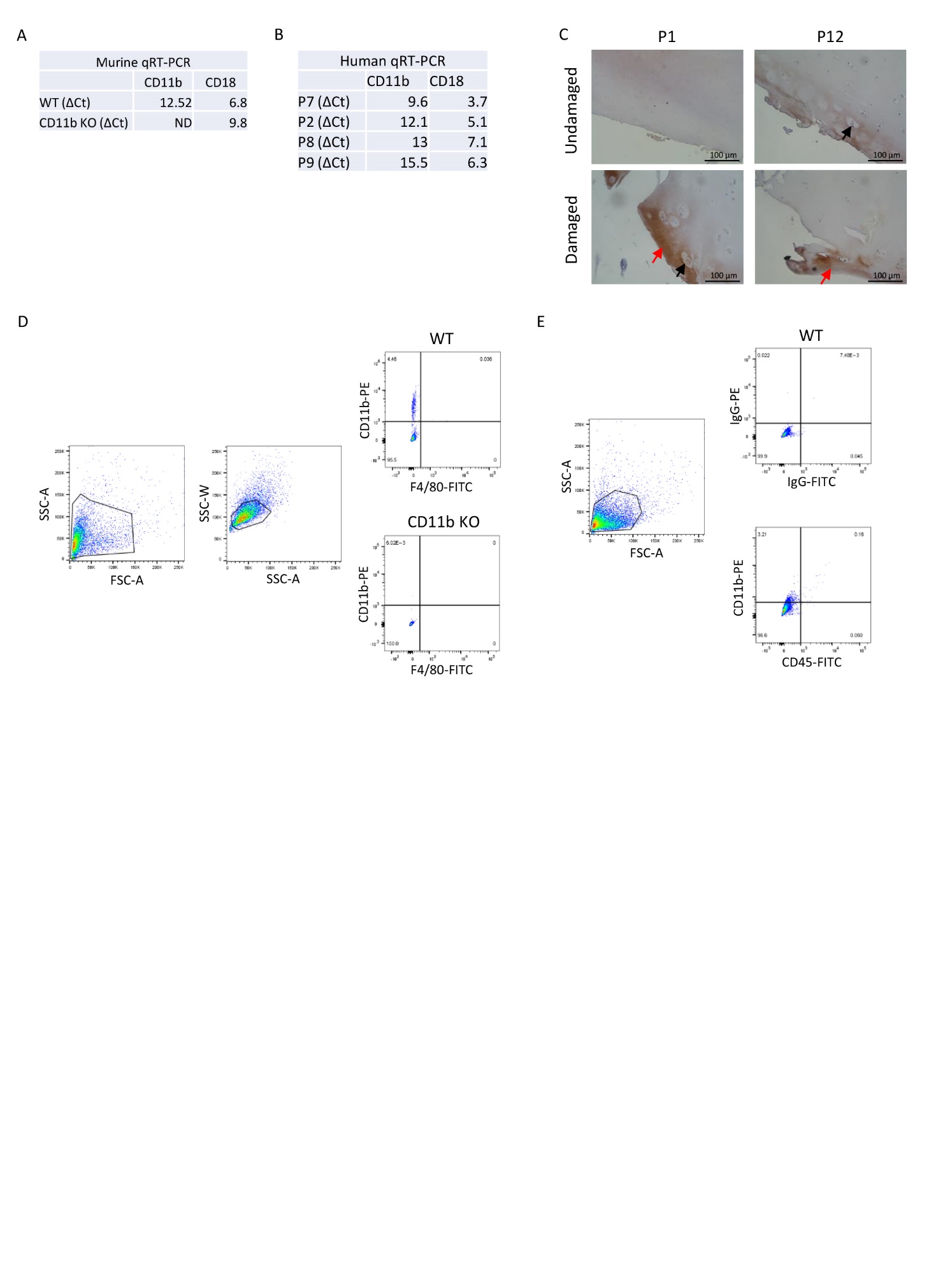


## Supplementary Figure 1. (A) qRT-PCR of the indicated genes in primary murine chondrocytes at basal level. Values represent ΔCt between the indicated genes and the housekeeping gene Gapdh. (B) qRT-PCR of the indicated genes in primary human chondrocytes from four OA patients at basal level. Values represent ΔCt between the indicated genes and the housekeeping gene Gapdh. (C) CD11b immunohistochemical staining (brown) in undamaged and damaged femoral knee cartilage from 2 OA patients undergoing joint replacement (black arrows indicate intracellular CD11b expression, red arrows indicate extracellular CD11b expression). Scale bars 100 μm. (D-E) Flow cytometry analysis of CD11b, F4/80 and CD45 expression on primary murine chondrocytes obtained from WT and CD11b KO mice.

**
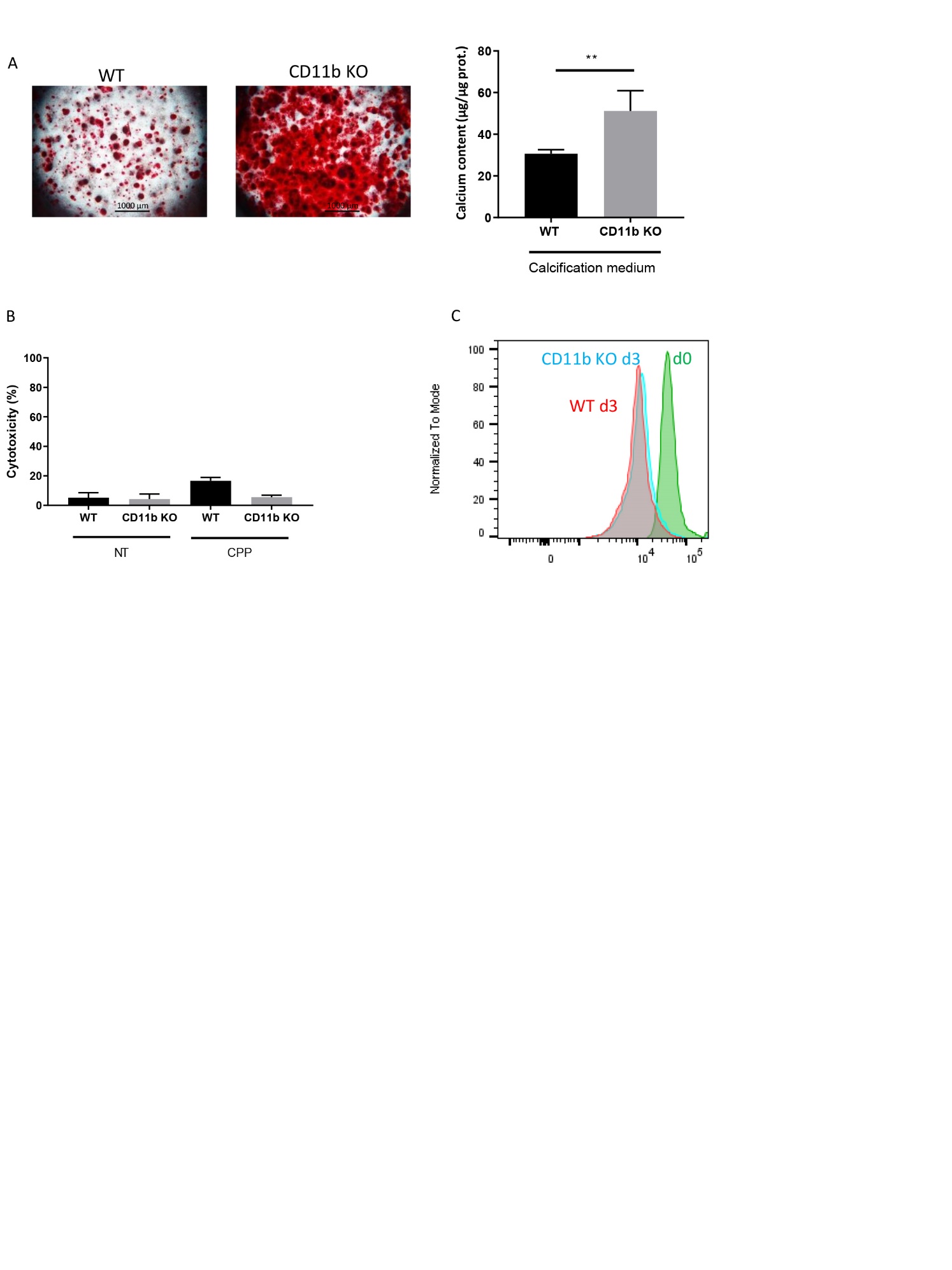
**

**Supplementary Figure 2.** **(A)** Alizarin Red staining of WT and CD11b KO primary murine chondrocytes stimulated with calcification media (50 µg/mL ascorbic acid, 20 mM β-glycerol phosphate) in complete Fitton-Jackson Modified (BGjb) medium (Gibco) + 10% FBS for 2 weeks. Pictures show one representative culture well of one experiment out of three independent experiments. The graph shows calcium content measured in μg per μg of proteins. Values represent means±SD of triplicates samples. ** p<0.01. Scale bars 1000 μm. **(B)** Percentage of cytotoxicity measured by quantification of Lactate Dehydrogenase (LDH) release by WT and CD11b KO chondrocytes, treated or not with secondary CPP. Values represent means±SD of triplicates samples. **(C)** Proliferation assay performed by FACS in WT and CD11b KO chondrocytes. Green curve represents proliferation of both cell type at day 0, red curve proliferation of WT chondrocytes at day 3, and blue curve proliferation of CD11b KO chondrocytes at day 3.


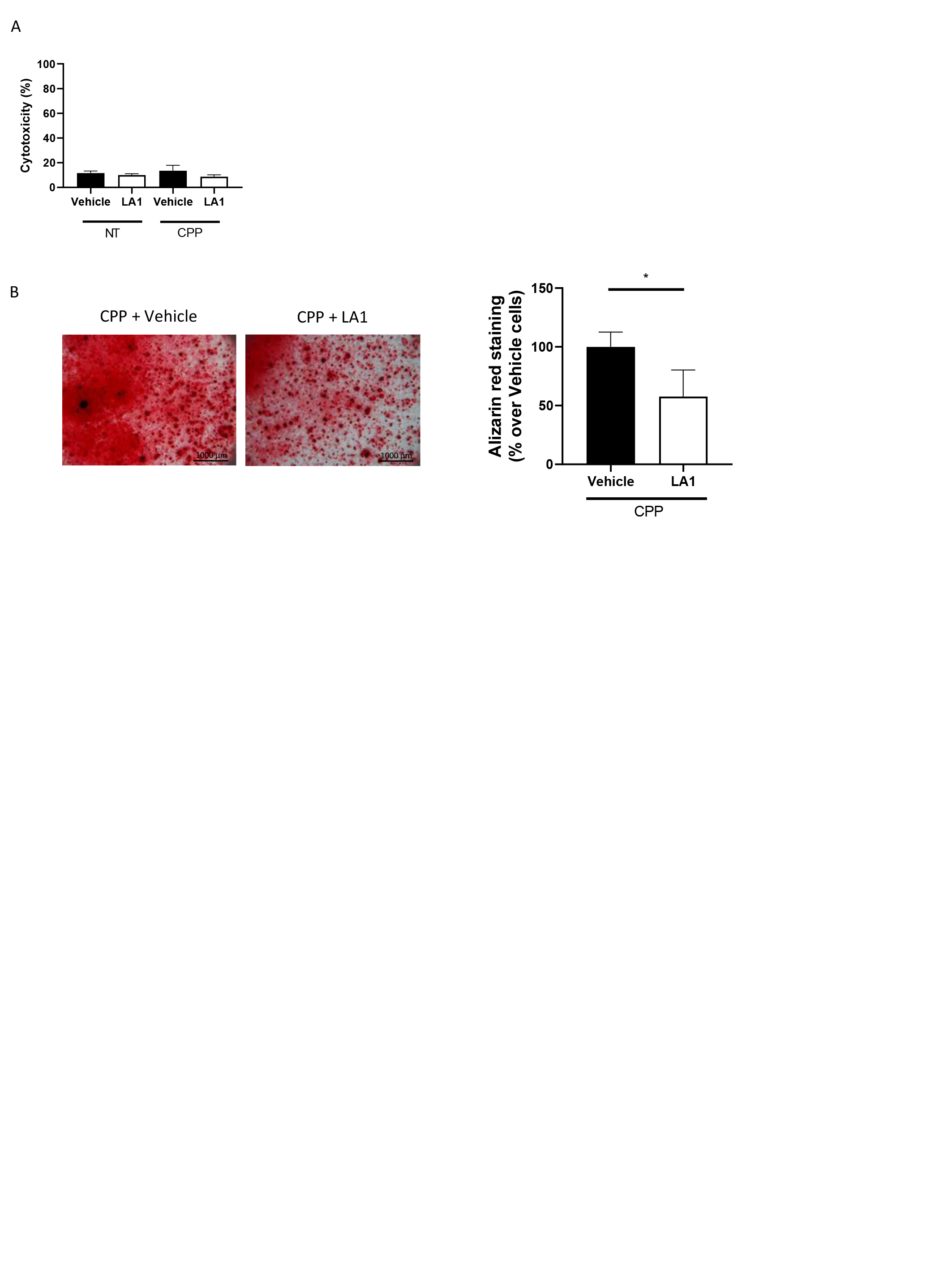


**Supplementary Figure 3.** **(A)** Percentage of cytotoxicity measured by quantification of LDH release by WT chondrocytes treated or not with LA1 and with secondary CPP for 24 h. Values represent means±SD of triplicates samples. **(B)** Alizarin Red staining of human primary synoviocytes stimulated with secondary CPP ± LA1 (20 μM) for 24 h in DMEM + 10% FBS. Pictures show one representative culture well of one experiment out of three replicates from P4. The graph shows the % of Alizarin Red positive surface over the total surface, normalized on Vehicle-treated cells. Values represent means±SD of triplicates. *p<0.05. Scale bars 1000 μm.


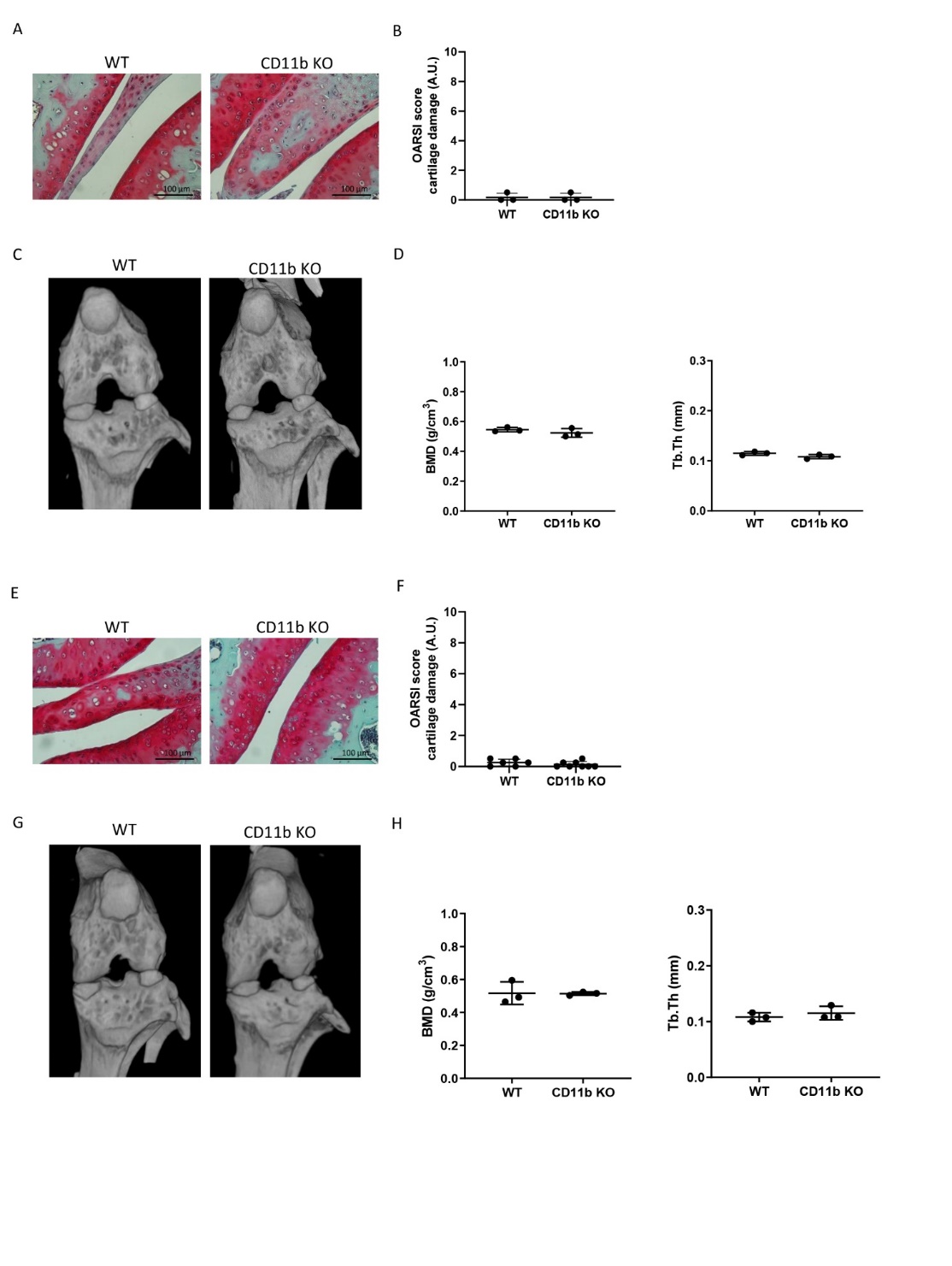


Supplementary Figure 4. (A) Representative Safranin-O histological staining of knee joints from sham-operated 20 weeks old WT and CD11b KO mice. Scale bars 100 μm. (B) Corresponding total OARSI score for cartilage damage in medial compartment of WT and CD11b KO knees. Values represent means±SD. (C) Representative micro-CT scan images of knee joints from sham-operated 20 weeks old WT and CD11b KO mice. (D) Corresponding CTAnalyzer quantitative analysis of tibial subchondral bone of WT and CD11b KO mice. Bone mineral density (BMD, g/cm^3^) and trabecular thickness (Tb.Th, mm). Data are expressed as the mean±SD. (E) Representative Safranin-O histological staining of knee joints from 35-37 weeks old WT and CD11b KO mice. Scale bars 100 μm. (F) Corresponding total OARSI score for cartilage damage in medial compartment of WT and CD11b KO knees. Values represent means±SD. (G) Representative micro-CT scan images of murine knee joints from 35-37 weeks old WT and CD11b KO mice. (H) Corresponding CTAnalyzer quantitative analysis of tibial subchondral bone of WT and CD11b KO mice. Bone mineral density (BMD, g/cm^3^) and trabecular thickness (Tb.Th, mm). Data are expressed as the mean±SD.
